# Composite Polyvinylpyrrolidone–Sodium Alginate—Hydroxyapatite Hydrogel Films for Bone Repair and Wound Dressings Applications

**DOI:** 10.3390/polym13223989

**Published:** 2021-11-18

**Authors:** Inna V. Fadeeva, Elena S. Trofimchuk, Anna A. Forysenkova, Abdulrahman I. Ahmed, Oleg I. Gnezdilov, Galina A. Davydova, Svetlana G. Kozlova, Aurora Antoniac, Julietta V. Rau

**Affiliations:** 1 Baikov Institute of Metallurgy and Material Science RAS, Leninsky, 49, 119334 Moscow, Russia; aforysenkova@gmail.com; 2 Department of High-Molecular Compounds, Lomonosov Moscow State University, GSP-1, 1-3 Leninskiye Gory, 119991 Moscow, Russia; elena_trofimchuk@mail.ru; 3 Department of Physics, Kazan Federal University, Kremlevskaya 18, 420008 Kazan, Russia; abdphysic82@yahoo.com (A.I.A.); goi@yandex.ru (O.I.G.); 4 Department of Physics, University of Al-Hamadaniya, Mosul 41001, Iraq; 5 Institute of Theoretical and Experimental Biophysics of RAS, Institutskaya 3, 142290 Pushchino, Moscow reg., Russia; davidova_g@mail.ru; 6 National Medical Research Center of Obstetrics, Gynecology and Perinatology, Academician Oparin Str., 117997 Moscow, Russia; 7 Department of Natural Science, Novosibirsk State University, Pirogova Street 2, 630090 Novosibirsk, Russia; s.kozlova@g.nsu.ru; 8 Department of Metallic Materials Science and Physical Metallurgy, University Politehnica of Bucharest, Street Splaiul Independentei, 060042 Bucharest, Romania; antoniac.aurora@gmail.com; 9 Istituto di Struttura della Materia, Consiglio Nazionale delle Ricerche (ISM-CNR), Via del Fosso del Cavaliere, 00133 Rome, Italy; 10 Department of Analytical, Physical and Colloid Chemistry, I.M. Sechenov First Moscow State Medical University, Trubetskaya Street, Build. 8/2, 119991 Moscow, Russia

**Keywords:** polyvinylpyrrolidone, sodium alginate, hydroxyapatite, composite films

## Abstract

Today, the synthesis of biocompatible and bioresorbable composite materials such as “polymer matrix-mineral constituent,” which stimulate the natural growth of living tissues and the restoration of damaged parts of the body, is one of the challenging problems in regenerative medicine. In this study, composite films of bioresorbable polymers of polyvinylpyrrolidone (PVP) and sodium alginate (SA) with hydroxyapatite (HA) were obtained. HA was introduced by two different methods. In one of them, it was synthesized in situ in a solution of polymer mixture, and in another one, it was added ex situ. Phase composition, microstructure, swelling properties and biocompatibility of films were investigated. The crosslinked composite PVP-SA-HA films exhibit hydrogel swelling characteristics, increasing three times in mass after immersion in a saline solution. It was found that composite PVP-SA-HA hydrogel films containing HA synthesized in situ exhibited acute cytotoxicity, associated with the presence of HA synthesis reaction byproducts—ammonia and ammonium nitrate. On the other hand, the films with HA added ex situ promoted the viability of dental pulp stem cells compared to the films containing only a polymer PVP-SA blend. The developed composite hydrogel films are recommended for such applications, such as membranes in osteoplastic surgery and wound dressing.

## 1. Introduction

Over the past decades, calcium phosphate (CP) materials have been successfully used in bone surgery [1,2,3,4,5,6,7]. The most widespread materials are based on hydroxyapatite (HA) and tricalcium phosphate, which is more resorbable with respect to HA. In addition to advantages such as high biocompatibility and corrosion resistance in the tissue environment, they have several drawbacks, such as fragility, a possible deformation and relatively low bioresorption rate with respect to new bone tissue growth [8]. In order to overcome these disadvantages, composite mineral-polymer materials have been developed [8,9].

The first attempts to obtain composite mineral-polymer materials for bone tissue engineering were based on trial modelling the processes that occur in the body during the formation of bone tissue [10,11,12]. This applies both to synthesis methods (mineralization of polymer fibers) and to the first polymers studied, such as collagen (type I and II) and gelatin [13,14,15,16]. Currently, the range of applied polymers significantly expanded—chitosan, starch, polyvinyl alcohol, polycaprolactone and several others have been applied [17,18,19,20]. Blends of natural and synthetic polymers act for a new class of materials with improved mechanical properties and biocompatibility compared to single-component materials [21,22,23].

Polyvinylpyrrolidone (PVP) is a hydrophilic, muco-adhesive polymer with good hemocompatibility and biocompatibility [24], which belongs to the “GRAS” group (Generally recognized as safe) by FDA (Food and Drug Administration, USA) [25]. Due to this, PVP has been used for many years as a broad-purpose biomaterial for blood substitutes [26], vitreous substitutes [27], as the main component for wound dressing coatings [28,29,30] and for the delivery of drugs and genes [31,32,33].

PVP hydrogels have limited application due to their poor mechanical properties. More often, mixtures of PVP with other polymers are used: collagen–PVP [34], tragacanth gum–PVP [35], PVP–sodium hyaluronate [36], PVP–chitosan [37] and polyvinyl alcohol–PVP [38]. Thus, the number of PVP hydrogels prepared from PVP mixtures plays a significant role in biomedical materials.

Sodium alginate (SA) is a linear polysaccharide derived from brown seaweed. It possesses highly negative charge densities, is water-soluble, nontoxic, biodegradable and biocompatible. SA hydrogels are prepared by chemical crosslinking in the presence of divalent and trivalent metal cations [39]. Moreover, SA is a biodegradable polymer with excellent gel-forming properties that is widely used in tissue engineering [40,41,42,43,44,45] and for the delivery and controlled release of drugs [46,47]. However, the use of SA hydrogels is limited, since SA is relatively inert when interacting with integrins of mammalian cells [48]; therefore, it is not adhesive for cells. In order to improve these properties, an interpenetrating hydrogel design looks promising [49,50,51].

The combination of favorable properties of each polymer component results in a new hybrid material with properties that are often significantly improved or differ from the properties of single polymers [52]. Therefore, the development of SA in combination with other polymers is a promising approach for improving bio-adhesiveness.

Regarding PVP and HA, the authors of [53] reported needle-like nanocrystals of HA precipitated on the PVP surface, mimicking natural body processes taking place during bone remodeling. The MTT colorimetric test for evaluating the metabolic activity of cells showed that the composites were biocompatible and can be used to fill in bone defects. In Reference [54], a two-layer material for wound dressings was obtained, and one of the layers was a composite of PVP-HA. Complete restoration of the skin was observed 4 weeks after implantation.

The purpose of the present study was to develop PVP-HA composite materials and to investigate its physico-chemical properties as well as to perform in vitro investigation of its biocompatibility. Moreover, in the present study, for the first time, composite PVP-SA-HA hydrogel films were developed, and their properties were investigated by a number of techniques, such as thermogravimetric analysis (TGA), X-ray diffraction (XRD), Fourier transform infrared spectroscopy (FTIR), nuclear magnetic resonance (NMR) spectroscopy, electron paramagnetic resonance (EPR), transmission and scanning electron microscopy (TEM and SEM) and swelling test. The cytotoxicity of the initial sample components was evaluated first by the standard MTT test by using mouse fibroblast NCTC clone L929. The adhesion characteristics and cytotoxicity of films were accessed by applying dental pulp stromal cells (DPSC)s by using the direct contact method and the Alamar Blue assay.

## 2. Materials and Methods

### 2.1. Synthesis of Composite Materials Based on Polyvinylpyrrolidone with Hydroxyapatite Obtained In Situ and Ex Situ

Solutions of Ca(NO_3_)_2_×4H_2_O (chemical grade, PanReacAppliChem, Barcelona, Spain), (NH_4_)_2_HPO_4_ (chemical grade, Chimmed, Moscow, Russia) and polyvinylpyrrolidone (PVP) Mw = 12 kDa (Boai NKY Pharmaceuticals Ltd., Tianjin, China) were used for the in situ synthesis of HA powders with PVP. Precipitation of calcium phosphates was performed at room temperature (20–25 °C) at a pH of ~11.5, in accordance with the Equation (1).

10Ca^2+^ + 6HPO_4_^2−^ + 8NH_3_ H_2_ → Ca_10_(PO_4_)_6_(OH)_2_ + 8NH^4+^ + 6H_2_ (1)

The in situ synthesis of the PVP with HA composite was carried out according to the procedure described in [55]. An aqueous solution was prepared by dissolving 7.18, 14.35 and 28.0 g of PVP in 200 mL of distilled water by stirring in order to obtain 2.76, 5.52 and 11.00 wt % concentration. After the formation of a homogeneous water-polymer mixture, 20 mL of 0.1 mol/L calcium nitrate solution was added to it. To regulate the reaction acidity, 20 mL of 25% NH_4_OH aqueous solution was added. Then, by stirring using a stirrer at 500 rpm, 20 mL of 0.06 mol/L diammonium phosphate solution was added drop by drop.

The PVP-HA ex situ composites were prepared as follows. HA ex situ was preliminary obtained by the deposition method from aqueous solutions. Calcium nitrate at 100 mL of 0.1 mol/L and 100 mL of 0.06 mol/L diammonium phosphate solutions were mixed by stirring, and 100 mL of 25% NH_4_OH aqueous solution were added. The obtained precipitate was filtered out by using a vacuum pump and dried at 100 °C for 7 days. Aqueous PVP solutions were prepared by dissolving 5.52, 11.00 and 22.00 g per 200 mL of distilled water by stirring. After the formation of a homogeneous water-polymer mixture, the powder of the obtained HA ex situ was added by stirring.

In order to reduce the solubility of films and to impart the film’s formation properties, sodium alginate (SA) (pure, Reakhim, Moscow, Russia) was mixed with PVP to obtain a blend of polymers SA and PVP. SA and PVP water solutions were prepared by dissolving 2.5 g of PVP and 2.5 g of SA in 95 mL of distilled water. An amount of 0.55 g of HA powder synthesized ex situ was dispersed in the polymer’s mixture by stirring by with a stirrer at 500 rpm.

#### Crosslinking of Composite PVP-SA-HA Films

For swelling and in vitro investigation, the composite PVP-SA-HA films were partially crosslinked by calcium chloride [56,57]. PVP-SA-HA films were immersed in 5 wt % solution of CaCl_2_ for one minute. At the same time, crosslinking of pure SA occurs through the formation of slightly soluble calcium alginate salt. PVP does not crosslink in this manner, but it is fixed by interlacing with the network of the crosslinked alginate.

The investigated samples are presented in Table 1.

The PVP-SA-HA composites films were formed on polypropylene substrates and dried at room temperature and humidity of 70 ± 10%. Bulk porous materials were obtained by foaming a reaction mixture synthesized as described earlier in [58] at a PVP concentration of 11.00 wt %. The foaming was performed by bubbling compressed air from the compressor through the mixture, as described in [59].

### 2.2. Thermogravimetric Analysis

Thermogravimetric analysis (TGA) of composites with different PVP and HA ratios was performed on the Netzsch STA 449F3 device (Selb, Germany) in an air atmosphere in the temperature range from 20 up to 700 °C at a speed of 10 °C/min. The data were processed using the NETZSCH Proteus software. For the TGA study, powders of composites of PVP-HA dried after the synthesis at 110 °C were used.

### 2.3. The Annealing

The annealing of bulk samples before X-ray, FTIR and specific surface area analyses was carried out for 1 h at 400 °C and 900 °C.

### 2.4. X-ray Phase Analysis

After heat treatment at 900 °C, X-ray phase analysis (XRD) was performed by using the DRONE 3M diffractometer (Saint Petersburg, Russia), CuKα radiation (*λ* = 0.154 nm). The average size of the crystallites was calculated by using the Scherrer formula (2):

(2) $$d\;=\;\frac{K\;\lambda }{\beta \;\cos\;\theta }$$

where *d* is the average size of the crystals (the region of coherent scattering), *K* is the dimensionless coefficient depending on the shape of the crystals, *λ* is the wavelength of X-ray radiation, *β* is the peak at full width half maximum (in radians on 2*θ* scale), and *θ* is the reflection angle (in degrees).

### 2.5. NMR Spectral Analysis

For sample 3 (see Table 1) and PVP, the MAS NMR spectra of ^1^H, ^31^P and ^13^C{^1^H} were recorded by using an AVANCE 400 WB NMR spectrometer (Bruker, Germany) with a MAS 4 BL CP BB DVT probe. The resonant frequency on proton nuclei was 400.27 MHz, 162.034 MHz on phosphorus nuclei and 100.613 MHz on carbon nuclei. The powder samples were tightly packed into a zirconium oxide rotor and spun to a rotation frequency of 7 kHz. The measurements were carried out at room temperature. The chemical shifts of the NMR signals of the samples were calibrated relative to the water signal (*δ* = 4.67 ppm).

### 2.6. FTIR Spectral Analysis

The FTIR spectra were obtained on the samples mixed with potassium bromide by means of the NicoletAvatar-330 (ThermoScientific, Waltham, MA, USA) spectrometer in the range of 400–4000 cm^−1^.

### 2.7. Scanning Electron Microscopy, Transmission Electron Microscopy and Specific Surface Area Analysis

The microstructure of composites was investigated on a Tescan VEGA3 scanning electron microscope (SEM) (Quorum Technologies Ltd., Great Britain, UK). Gold was previously sprayed onto the dry sample’s surface by using a Q150R Plus rotary pumping spraying unit (Quorum Technologies Ltd., Great Britain, UK). Transmission electron microscopy (TEM) images and electron diffraction patterns were obtained by using the LEO 912 AB OMEGA microscope (Carl Zeiss, Oberckochen, Germany). For this purpose, ultra-thin sections of PVP-HA composites prepared by ultramicrotomy with a diamond knife were placed on a copper mesh covered with a collodion substrate. The determination of interplane distances from electronograms was performed by using a standard gold sample. Coherent scattering region (CSR) calculations were performed according to the procedure described in Reference [60].

### 2.8. Specific Surface Area Analysis

The specific surface area of powders obtained from PVP-HA composites (samples 1–3, Table 1) after heat treatment at T = 400 °C was measured by a specific surface analyzer, TriStar 3000 V6.03A. N_2_ was used as adsorptive, and the bath temperature was 195.8 °C. The specific surface area was calculated according to the Brunauer–Emmett–Teller (BET) method according to the following formula (3):

(3) $$\frac{p/p_{0}}{a\left[\left(p/p_{0}\right)\right]}\;=\;\frac{C-1}{a_{m}C}\left(\frac{p}{p_{0}}\right)\;+\;\frac{1}{a_{m}C}$$

where:

$\frac{p/p_{0}}{a\left[\left(1-p/p_{0}\right)\right]}$—the ratio between system pressure and condensation pressure.

*a*—adsorption value;

*a_m_*—the volume of the monolayer on the surface of the adsorbent.

*c*—the ratio between the adsorption equilibrium constants in the first layer and the condensation constants.

### 2.9. Electron Paramagnetic Resonance Investigations

Electron paramagnetic resonance (EPR) measurements were performed on a Bruker Elexsys E680 spectrometer (Karlsruhe, Germany) in pulsed mode using the Hahn sequence of the following: *π*/2 − *τ*—*π* − *τ*—(electron spin echo—ESE), where the duration of *π*/2 is equal to 64 ns and *τ* = 250 ns. Registration of EPR spectra was performed by detecting the ESE integral intensity dependance on the magnetic field. The choice of the high-frequency range of the experimental equipment (W-band, microwave frequency is of 94 GHz) was justified by the need to achieve a higher spectroscopic resolution (that allows identifying distinct EPR signals with close g-factors) and high sensitivity (to register weak EPR absorptions). A helium flow cryostat was used for measurements at low temperatures (*T* < 297 K). Stable photo-induced paramagnetic centers were formed under laser radiation in continuous-wave modes with a wavelength of *λ* = 266 nm (ultraviolet light—UV).

### 2.10. Swelling Properties Investigation

The swelling data of crosslinked composites with different HA content were obtained by samples weighing after immersion in the 0.9% NaCl solution. Weighing was carried out by electronic weight scales (VМ 510D, OKB Vesta, Saint-Petersburg, Russia).

### 2.11. In Vitro Studies

The cytotoxicity of the initial sample components was evaluated by using a standard MTT test with mouse fibroblast NCTC clone L929 [61]. The cells were cultured in a Dulbecco’s Modified Eagle’s culture medium (DMEM) containing 10% FBS (HyClone) (Waltham, Massachusetts, USA), 100 units/mL of penicillin/streptomycin and 2 mM of glutamine in a CO_2_-incubator (5% CO_2_ atmosphere, 37 °C).

The viability of cells seeded on the sample surface was evaluated by means of the Alamar Blue assay [62], in which the resazurin compound (7-hydroxy-3H-phenoxazine-3-on-10-sodium salt oxide) (Sigma-Aldrich, Darmstadt, Germany) is reduced by enzymes of living cells to a fluorescent resorufin. The test samples were placed in the wells of the 24-well plate. DPSCs were sown in wells on the surface of samples with a density of 40,000 cells/cm^2^. Three holes were left free of cells and samples in order to measure the background recovery of resazurin. Afterwards, the plate with the cells was incubated in a CO_2_ incubator for 24 h under standard conditions. After that, 22 µL of resazurin was added to each well to reach a final concentration of 50 µM. The plates were incubated for 3 h. The fluorescence of the reduced dye was determined by using a flatbed spectrofluorimeter Tecan Infinity F200 (Tecan, Männedorf, Switzerland) (*λ*_excitation_ = 530 nm, *λ*_emission_ = 590 nm).

In order to determine the cytotoxicity of materials, the direct contact method was applied. For this purpose, primary mesenchymal stromal stem cells were extracted from the pulp of the human third molar and removed according to orthodontic indications. The cell culture was used on the fourth passage. Film samples were placed in the wells of the 24-well plate, and DPSCs with a density of 40,000 cells/cm^2^ were seeded on their surface. The viability of cells was evaluated by using the Axiovert 200 microscope (Carl Zeiss, Oberkochen, Germany). The cells were investigated after 24 h. For the analysis, the method of fluorescent cell staining was used, using fluorescent dye SYTO 9 and propidium iodide. The DNA and RNA of living and dead cells were colored in green by the fluorescent dye SYTO 9 (at *λ*_excitation_ = 450–490 nm, *λ*_emission_ = 515–565 nm), whereas the nuclei of dead cells were colored in red by propidium iodide (at *λ*_excitation_ = 546 nm, *λ*_emission_ = 575–640 nm).

In order to obtain cell viability and survivability data, each point was measured in 8 repetitions. The standard deviation was calculated for each point in all sections. The statistical analysis of the reliability was performed according to the Mann–Whitney U-test (*p* ≤ 0.01).

The standard NCTC L929 fibroblast cell line of murine subcutaneous connective tissue was provided by the Institute of Cytology of the Russian Academy of Sciences in Moscow, Russia, from their collection of cell cultures.

## 3. Results and Discussion

### 3.1. TGA Investigation

**Figure 1:**
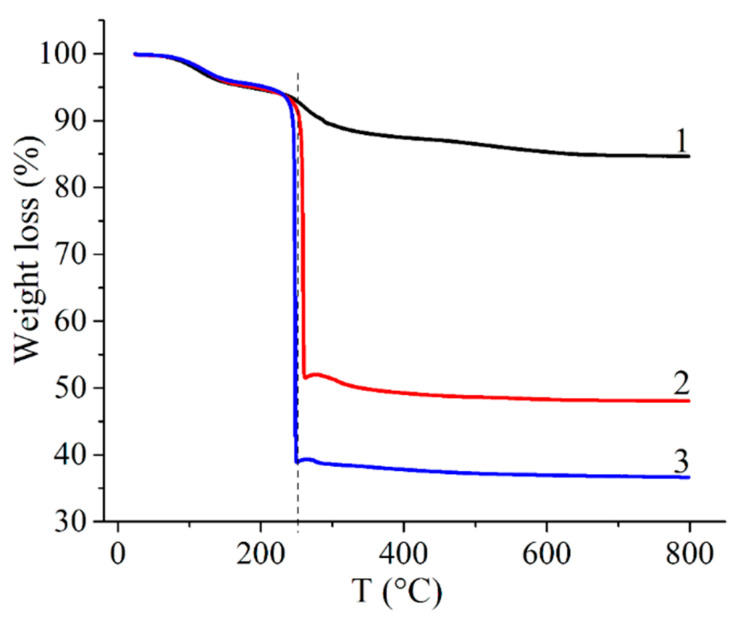
TGA curves of PVP-HA composites: 1—sample 1; 2—sample 2; 3—sample 3 (Table 1, samples 1–3, respectively).

First, PVP-HA composites with HA synthesized in situ were studied. The behavior of the composites, prepared at different PVP and HA ratios, during high-temperature annealing was investigated by the TGA method (Table 1, samples 1–3). As observed in Figure 1, all samples lose mass in two stages. The mass decrease in the temperature range of 30–250 °C is probably due to the loss of free and bound (crystallization) water from the composites and transformation of the amorphous precipitated HA into a crystalline one. At the second stage, in the range of 250–800 °C, thermal-oxidative destruction of the polymer matrix and further crystallization of HA occurs. A similar behavior of PVP-HA (HA added ex situ) composite during temperature treatment upon TGA was described in Reference [63]. At temperatures higher than 500 °C, the sample’s mass remains unchanged. The mass fraction of precipitates after heating was 85%, 48% and 37% for samples 1–3 (Table 1).

### 3.2. X-ray Analysis

**Figure 2:**
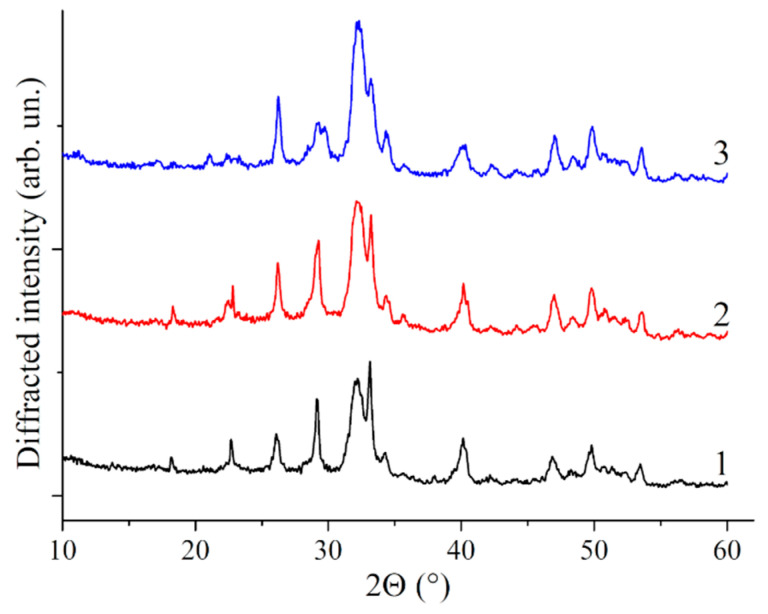
X-ray diffraction patterns obtained after the annealing of the composites at 900 °C. 1—sample; 2—sample 2; 3—sample 3. All peaks correspond to HA (card #9-432) (samples 1–3, Table 1).

In our case, the obtained samples are X-ray amorphous since aging of precipitate was not performed, as it was carried out by microwave treatment in Reference [64]. The analysis of bacterial cellulose-HA composites without preliminary heat treatment also did not register peaks related to HA [65]. Therefore, X-ray analysis of the PVP-HA composites confirmed that, after heat treatment at 900 °C, the HA phase is registered for all the investigated compositions (see Figure 2).

### 3.3. NMR Analysis

**Figure 3:**
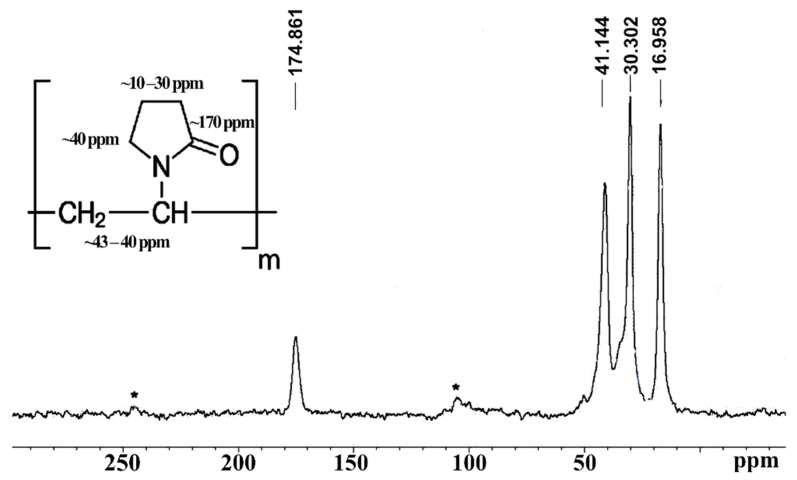
The NMR spectrum of MAS ^13^C{^1^H} for the PVP polymer. *N*-Рolyvinylpyrrolidone structure (insert). *—satellites from the rotation of the sample at a frequency of 7 kHz (sample 3).

The most indicative sample for the characterization of the obtained samples is the MAS ^13^C{^1^H} NMR spectra for the initial PVP polymer (Figure 3) and the mixture of the PVP polymer with HA (it has the same spectral pattern as that shown in Figure 3). The obtained spectrum demonstrates the presence of characteristic NMR signals from PVP (insert in Figure 3).

The ^31^P NMR spectrum of the PVP-HA sample 3 (see Table 1) is a single line with a chemical shift of 3 ppm that indicates that all phosphorus nuclei from HA remain in the same chemical environment. The ^1^H NMR spectrum of a PVP polymer consists of one unresolved NMR signal at 2.5 ppm. The NMR spectrum of the PVP-HA sample consists of two NMR signals at 2.5 ppm and −4.8 ppm. The integral intensity of the NMR signal at −4.8 ppm is significantly less than the intensity of the NMR signal at 2.5 ppm, and this NMR signal is probably associated with water adsorbed in the pores of HA. In general, NMR data are consistent with X-ray diffraction patterns.

### 3.4. FTIR Analysis

**Figure 4:**
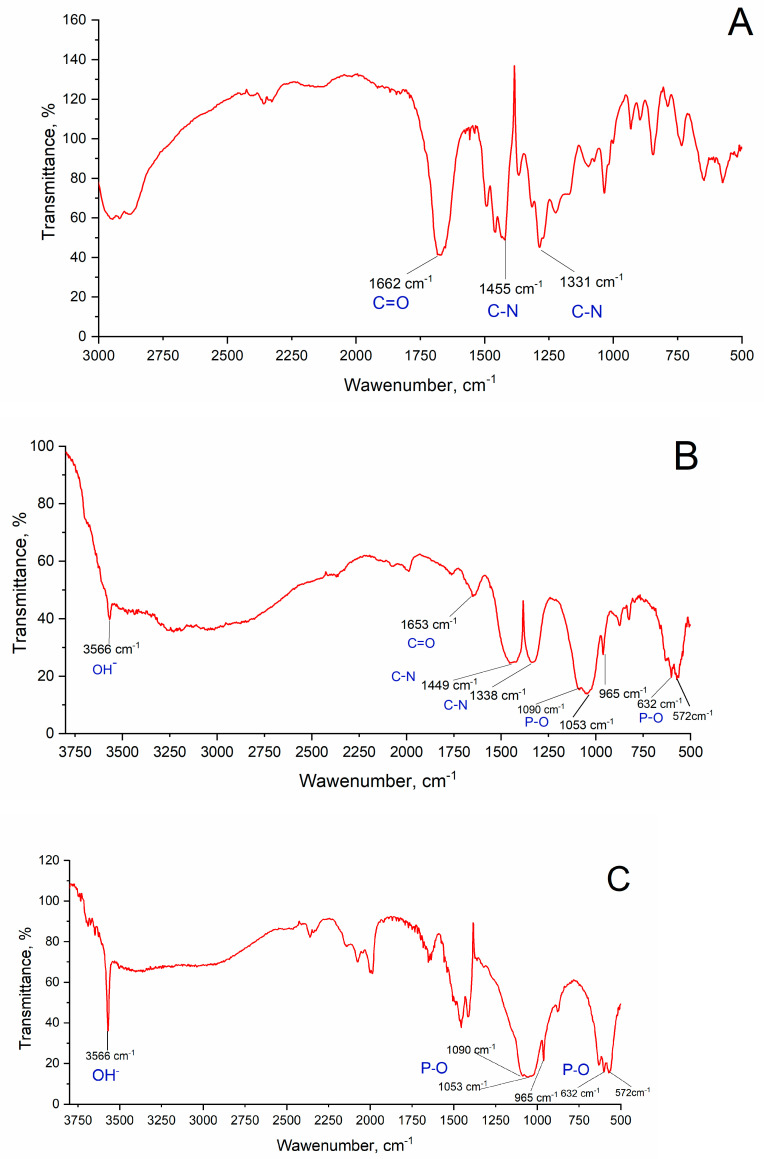
IR spectra of (**A**) PVP, (**B**) composite sample 3 (Table 1) and (**C**) sample 3 after annealing at 400 °C.

In Figure 4A, the IR spectrum of PVP is presented. In the spectra of sample 3 (Table 1) (Figure 4B), it is possible to distinguish the bands characteristic of PVP and HA, despite the overlap of some PVP bands with the phosphate group ones. The most intense band at 1653 cm^−1^ corresponds to the valence vibrations of the carbonyl group (C=O) [66,67]. Triplets of valence vibrations at 1090, 1053 and 965 cm^−1^ and at 632, 572 and 472 cm^−1^ correspond to the PO_4_^3−^ group in HA [67]. An intense band at 3566 cm^−1^, assignable to the OH^−^ group, indicates HA formation. These data correspond to those presented in References [63,64]. After heat treatment at 400 °C (Figure 4C), the PVP bands completely disappear, which is caused by PVP thermal decomposition.

### 3.5. Dimensional Analysis

**Figure 5:**
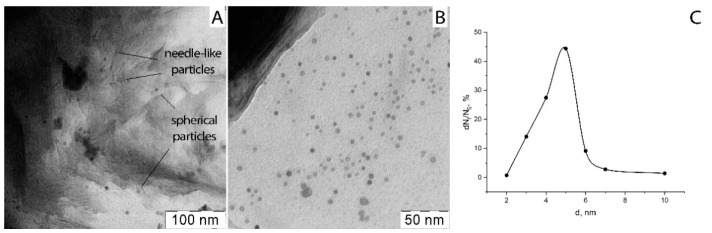
(**A**, **B**)—TEM microphotographs of ultra-thin sections of composite PVP-HA (sample 1, Table 1) at different magnifications; (**C**)—HA particle size distribution.

After the synthesis, the obtained PVP-HA in situ composites were X-ray amorphous. Additionally, their structure was investigated by TEM. The TEM microphotographs of ultra-thin sections are presented in Figure 5. The spherical nanoparticles with an average diameter of about 5 nm (Figure 5C), as well as their agglomerates with a size of about 100–200 nm, and needle-like particles with a thickness of several nanometers and a length of 20 nm were detected. Particle size and the formation of agglomerates in PVP-HA composites are affected by PVP content, as shown in [68]. HA formation in the PVP solution according to reaction (1) takes place in the following manner: ions of calcium interact with phosphate ions, resulting in the formation of amorphous calcium phosphate, and each of its molecule is surrounded by PVP chains [69].

**Figure 6:**
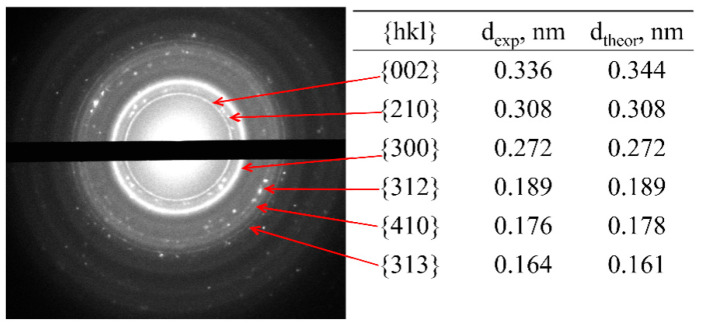
(**left**) Electron diffraction pattern of the sample 1 (Table 1). (**right**) Corresponding experimental (*d*_exp_) and theoretical (*d*_table_) [69] interplanar spacings.

The highly sensitive method of electron microdiffraction shows that these composites (sample 1, Table 1) include a calcium phosphate phase. The electron diffraction pattern (Figure 6) was obtained from the sample area shown in Figure 5A, evidencing point reflexes from a system of randomly arranged small single particles that form ring reflexes inherent in isotropic systems. At the same time, it was noted that needle-like particles are X-ray amorphous and do not exhibit reflexes on the electron diffraction pattern. The corresponding dark-field image shows that only spherical particles and their agglomerates contribute to the diffraction pattern, indicating the amorphous state of the needle-like particles. For the obtained crystal reflexes, the interplanar distances were calculated (table in Figure 6), and their correlation with the Miller crystallographic indices was performed. It turned out that the crystal phase in the samples can be attributed to the HA (Ca_10_(PO4)_6_(OH)_2_) phase with a hexagonal crystal lattice.

Table 2 contains the results of the determination of dimensional characteristics of the prepared materials by various methods. The PVP-HA composites were heat treated at 400 °C in order to obtain powders for dimensional characteristics determination.

In PVP-HA composites, the CSR values calculated from obtained XRD data with the Scherrer Equation (1) (17–20 nm) exceeded the particle size obtained from TEM data (30–45 nm) due to the presence of an organic component in the composite. The CSR of the investigated composites does not depend on the initial ratio of their components.

### 3.6. EPR Analysis

The initial samples of HA, PVP and the PVP-HA composite (sample 1, Table 1) were studied by a high-frequency EPR spectroscopy. In the HA sample and in the temperature range of 100–300 K, no EPR signals were detected since the materials do not contain paramagnetic impurities (such as various complexes of 3D metals embedded either into the calcium phosphate structure [70,71,72] or located on their surfaces [73]) at least within the sensitivity of the spectrometer (<10^13^ spin/g). Consequently, the studied materials can be considered as EPR silent.

**Figure 7:**
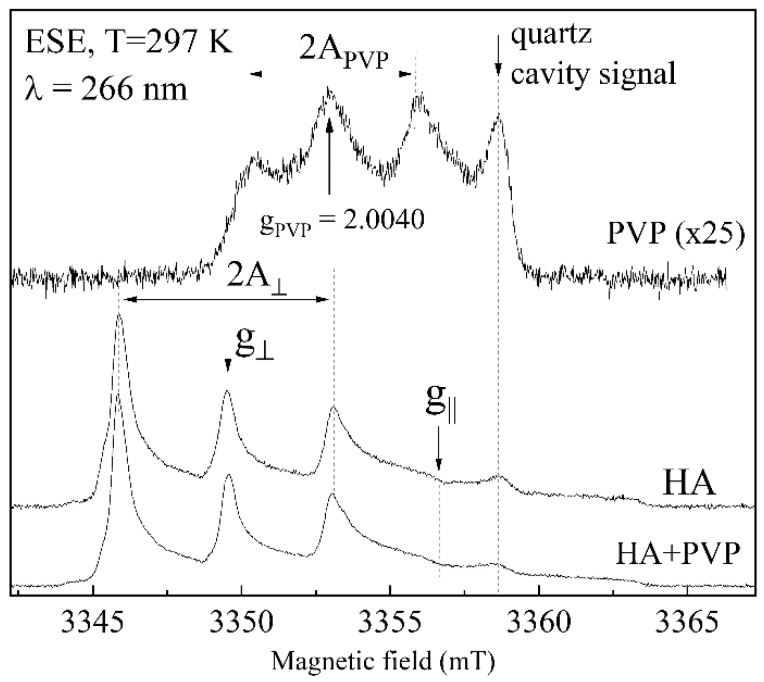
Pulsed EPR spectra for PVP (upper curve), HA (middle curve) and sample 1 (lower curve) under UV radiation (*λ* = 266 nm) at room temperature in the W-band. Signal from PVP is shown at much higher enhancement (25×) compared to HA and sample 1. Signal arising from the quartz tube is labelled.

However, under UV irradiation, it was possible to record the EPR spectra. Photo-induced EPR signals can be directly generated in the EPR cavity by using low-power lasers. As it turned out, even at room temperature under an UV laser radiation, stable paramagnetic centers can be created (see Figure 7), and they disappear after several hours after switching off of the UV laser.

The EPR spectra of the irradiated HA and of the PVP-HA composite are mainly defined by the presence of the impurity of nitrates (from ammonium nitrate, which is a by-product of the HA synthesis according to reaction (1)) transferred into the NO_3_^2−^ stable radicals of axial symmetry under the influence of UV-irradiation with the spectroscopic parameters of g_||_ = 2.0025, g = 2.006, A_||_ = 6.7 mT and A = 3.4 mT. According to Reference [74], the extracted EPR parameters correspond to NO_3_^2−^ in B position (i.e., phosphate site) in the HA structure. From the EPR results, it follows that the introduction of PVP has little effect on the NO_3_^2−^ EPR spectrum, resulting only in a slight inhomogeneous broadening of the peaks. This means that the crystal structure of HA (at least in the environment of the nitrate ion) is not influenced much by the presence of PVP. No carbonate radicals, such as CO^2−^, CO^3−^ and CO_3_^3−^ (often found in the irradiated calcium phosphates [75]), were detected by the applied technique that confirms the carbonates’ absence in HA.

The signal from UV-irradiated PVP is very weak compared to the signal from the stable nitrogen radical in HA (Figure 7); moreover, according to Frunze and Berlin [76], this is mainly due to the isotropic hyperfine interaction of carbon-centered defects (g_PVP_ = 2.0040) with the polymer ^14^N nuclei (I = 1, therefore 2I + 1 = 3 lines) with A_PVP_ = 2.75 mT. This weak signal is masked by the intensive NO_3_^2−^ response in the PVP-HA composite. A spurious signal labelled in Figure 7 belongs to the quartz tube and the EPR cavity.

### 3.7. Microstructural Analysis

**Figure 8:**
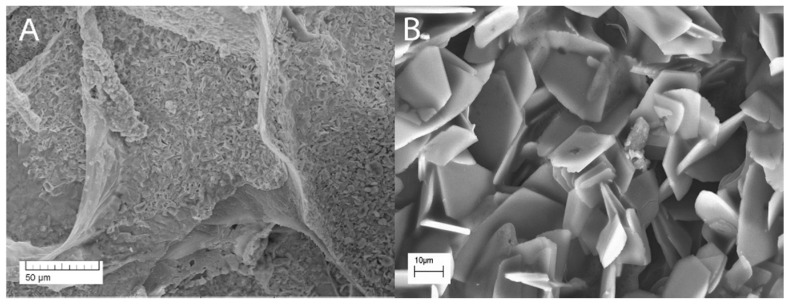
Microstructure of the composite PVP-HA material (sample 5, Table 1). Magnification: (**A**)—200×; (**B**)—2000×.

The microstructure of the composite material PVP-HA ex situ (sample 5, Figure 8) is fairly uniform and contains irregular plate particles with at a microscale size (10–20 µm).

### 3.8. The Crosslinking of Composite PVP-SA-HA Film

As it was mentioned in Section 2.1., in order to improve PVP-HA composite film-forming properties [56], SA was added, and several samples were prepared (see Table 1, samples 6–11) and crosslinked. The need for this is due to the fact that PVP-SA-HA films without crosslinking are instable in water media. Crosslinking was carried out in order to reduce their solubility, since SA forms a slightly soluble calcium salt in the CaCl_2_ solution.

A comparison of solubility of non-crosslinked PVP-SA-HA film, crosslinked PVP-SA-HA film and crosslinked SA film was made. Non-crosslinked film dissolved in 0.9 wt % NaCl solution during 15 min. Pure SA film lost its structure and formed a flake-like precipitate after 6 h of exposition in the 0.9 wt % NaCl solution. The PVP-SA-HA composite film kept its form at least during 5 days, but some weight loss was detected. The film lost up to 15% of mass during the first 6 h, and after that the mass was practically stable during the next 24 h; after the next 5 days, the mass of the film was unchanged.

The form retention of the PVP-SA-HA film сan likely be explained by the formation of PVP and SA interpenetrating networks. The initial loss of film mass seems to be associated with the transition into the solution of PVP and SA chains poorly fixed at the film surface.

Earlier [77], crosslinking of the methylcellulose-SA films with iron ions was investigated. The methylcellulose-SA films crosslinked with iron(III)-salicylic acid complex were stable for 14 days.

Thus, the crosslinked blend of PVP-SA has better stability in the solution compared to the crosslinked pure SA. However, additional studies of the Сa-crosslinking conditions’ effect on the solubility and other properties of films are needed.

### 3.9. The Swelling Data

Since the addition of SA to the PVP-HA composite resulted in a significant improvement of the stability of prepared composite film materials, further investigations, including biological properties, were performed on materials containing SA. Swelling properties were investigated for the PVP-SA polymer blend and the PVP-SA-HA ex situ (sample 6 and 8, Table 1, respectively).

**Figure 9:**
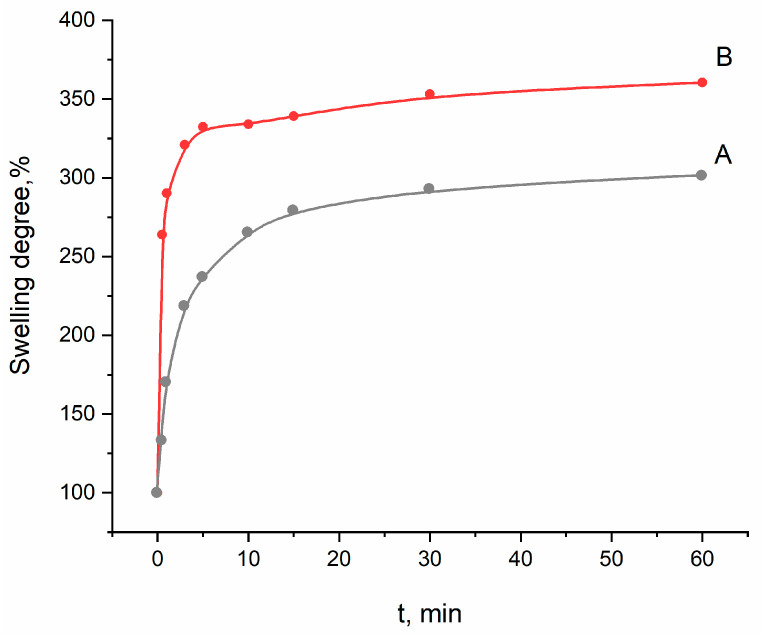
Swelling curve of sample 8 (PVP-SA-HA)—A; sample 6 (PVP-SA)—B.

After immersion in the saline solution, the samples started to swell, which was accompanied by their increase in mass (Figure 9). The highest increase was observed during the first 10 min; during the next 50 min of the experiment, the mass of the films practically did not change. The film samples immersed in the saline solution were stable and maintained during 21 days. Similarly, the process of swelling occurred in the case of films based on methylcellulose and SA [77]. It should be noted that swelling is a beneficial property for wound dressing materials. As observed from Figure 9, the composite PVP-SA-HA films are characterized by approximately 3 times increase in mass. This property could be used to impregnate the developed composite hydrogel films with drugs and antimicrobial agents for wound treatment.

### 3.10. In Vitro

**Figure 10:**
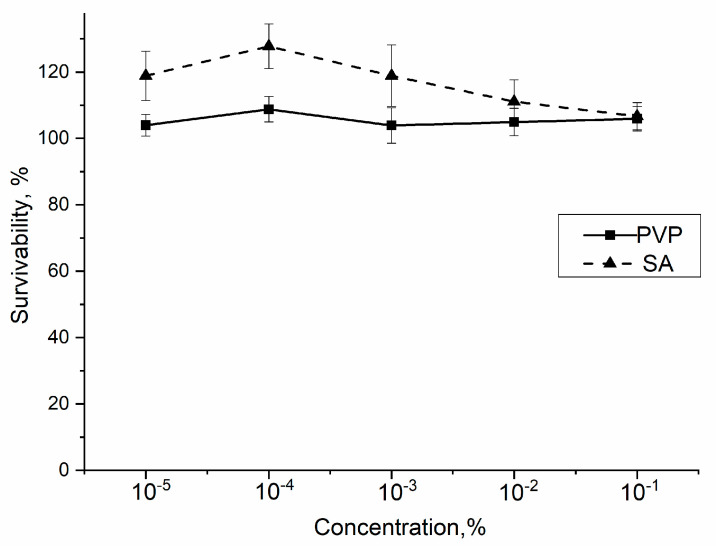
The viability of mouse fibroblast NCTC clone L929 upon addition of PVP and SA. The control sample corresponds to 100%. The statistical analysis of reliability was performed according to the Mann–Whitney U-test (*p* ≤ 0.01).

First of all, the effect of single component—PVP—on standard mouse fibroblasts NCTC clone L929 viability was determined by using the MTT test. It was shown that PVP in concentrations of 10^−5^–10^−1^ mass% did not have a statistically significant effects on the mouse fibroblasts incubated for 24 h (see Figure 10).

At PVP concentrations of 10^−5^–10^−1^ mass%, the number of cells did not decrease after 24 h with respect to the control, and even an increase in the viability of the NCTC was detected. Therefore, in the investigated range of concentrations, PVP is cell friendly.

According to the literature, SA is known to be non-cytotoxic [78].

**Figure 11:**
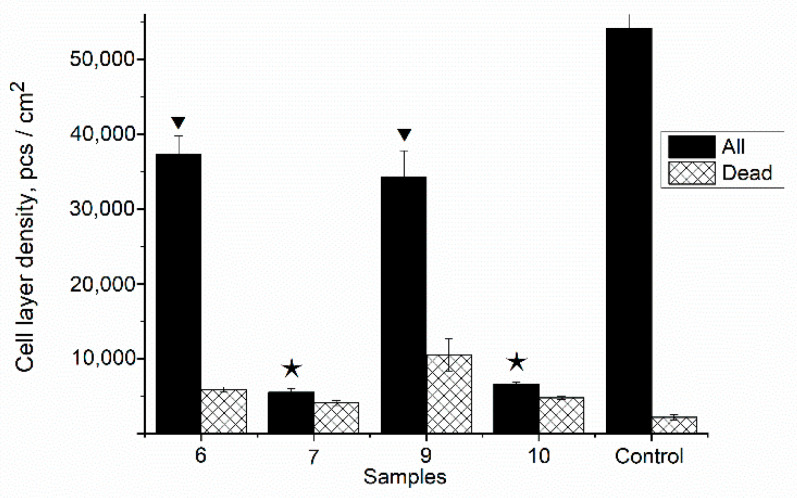
The number of DPSC mesenchymal stem cells cultured on the film surface. Sample 6—PVP-SA (1:1); sample 7—PVP-SA-HA (1:1, HA in situ); sample 9—PVP-SA (1:2); sample 10—PVP-SA-HA (1:2, HA in situ) (see Table 1). Control sample—glass slide. The triangles correspond to PVP-SA blends, the stars correspond to composites PVP-SA-HA in situ. The statistical analysis of the reliability was performed according to the Mann–Whitney U-test (*p* ≤ 0.01).

In order to clarify the effect of the prepared composite film samples with HA in situ on the DPSC mesenchymal stem cell proliferation in vitro, the composite samples 7 and 10 were studied in comparison with the polymer matrix PVP-SA with various PVP:SA’ ratios (sample 6 and 9, Table 1). The results of the direct contact method are presented in Figure 11. As it was shown, the addition of HA in situ (sample 7 and 10, stars, Figure 11) causes acute cytotoxicity of samples despite the composition of the polymer matrix. The cell layer density (for all cells) on the surface of the PVP-SA samples 6 and 9 without HA (triangles, Figure 11) has statistically significant differences from the one on the surface of the control sample, demonstrating that alginate induces a decrease in the ability of cells to form a dense homogeneous layer. This is confirmed also by the fact that the amount of dead cells increased with the increase in alginate content.

**Figure 12:**
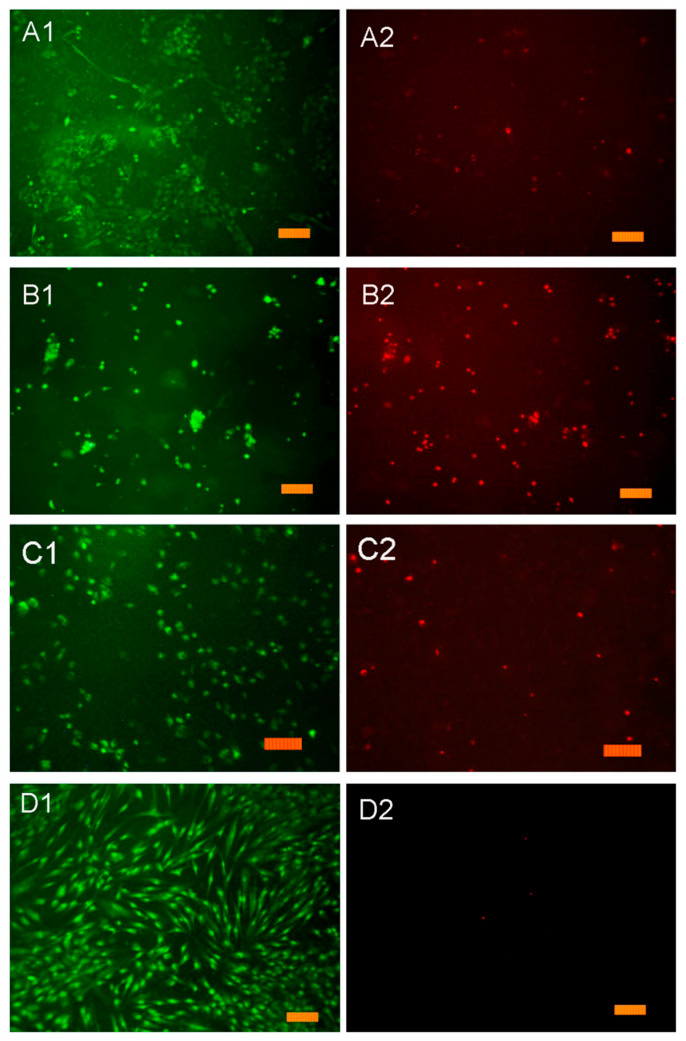
Resazurin test. DPSCs cultured for 24 h on the surface of composite film samples: (**A1**,**A2**)—sample 6; (**B1**,**B2**)—sample 7; (**C1**,**C2**)—sample 8; (**D1**,**D2**)—control sample and glass slide. The label corresponds to 100 µm.

The results of the resazurin test with DPSCs are shown in Figure 12. On the surface of the PVP-SA film without HA (sample 6), the normal morphology of DPSC cells is observed, but the density of the cell layer is lower than in the control sample (glass slide), which is probably due to the fact that the film contains SA. It was observed that, on SA’s surface, cells do not spread out but gather into spheroids. Previously, it was shown that cells retain a spherical shape when in contact with alginate hydrogels [79]. This fact also explains the data presented in Figure 11. The cell layer density on samples 6 (PVP:SA 1:1) and 9 (PVP:SA 1:2) has statistically significant differences from the cell layer density on the control. This is because cells’ spheroids on the surface of samples with alginate do not form a dense layer, which is confirmed also by the authors of Reference [80].

When DPSCs are cultured on the surface of the composite samples containing HA synthesized in situ (sample 7, Table 1) (Figure 11 and Figure 12), some of them die. This experimental evidence can be explained by the fact that when HA is formed in situ, reaction by-products, such as ammonia and ammonium nitrate, are released into the polymer solution (see Equation (1)). These chemical compounds are cytotoxic, and their presence results in cell death during cultivation. The increase in PVP concentration in the composite film improves the adhesion of cells and reduces the toxic effects of HA added in situ. In the composite samples containing HA synthesized ex situ (sample 8, Table 1), a large number of well spread cells were observed on the composite film’s surface, and the non-viable cells were practically absent; therefore, this indicates the absence of cytotoxic effects of the prepared composite films.

The cytotoxic effect of the PVP-SA-HA in situ samples determined by the direct contact method (Figure 11) is fully correlated with the data obtained in the resazurin test (Figure 12).

**Figure 13:**
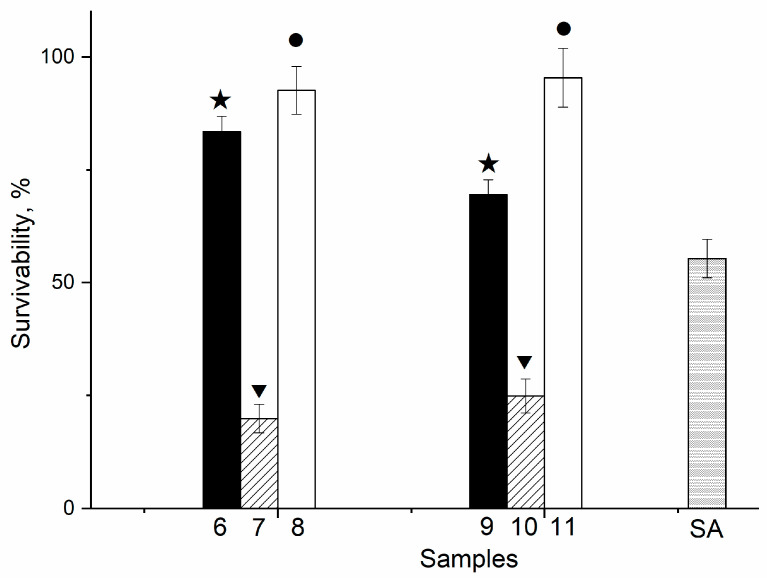
The number of DPSC cultured on the film surface of samples 6−11 (Table 1). Control sample (glass slide) corresponds to 100%. The stars correspond to PVP-SA blends, the triangles—to composites PVP-SA-HA in situ, the circles—to composites PVP-SA-HA ex situ. The statistical analysis of the reliability was performed according to the Mann–Whitney U-test (*p* ≤ 0.01).

In Figure 13, the number of the DPSCs cultured on the surface of film samples is shown. As one can observe, there is a decrease in the DPSCs viability on the PVP-SA-HA in situ films (samples 7 and 10) due to toxic by-products. The viability of cells on the surface of the PVP-SA-HA ex situ (samples 8 and 11) is much higher (*p* ≤ 0.01) compared to PVP-SA films (samples 6 and 9) and to the SA alone. Therefore, the PVP-SA-HA ex situ composite film samples are recommended for application as membranes in osteoplastic surgery and wound healing.

In Figure 13, the survivability of the DPSCs cultured on the surface of various film samples is shown. In this figure, the influence of the polymer matrix composition (samples 6 and 9, stars) and of the HA synthesis (in situ (samples 7 and 10, triangles) and ex situ (samples 8 and 11, circles)) on DPSC proliferation can be observed. The cell survivability for sample 6 is statistically significantly higher compared to sample 9 and to the pure alginate (83%, 69% and 55%, respectively). The samples with HA in situ (samples 7 and 10) decreased the survivability of cells to a level of 25%, regardless of the polymer matrix composition. Such an acute cytotoxicity is due to the presence of by-products of HA synthesis in their composition—ammonium and ammonium nitrate. On the other hand, there is an almost complete cell survival on the surface of the samples with HA ex situ (samples 8 and 11), and the corresponding survivability values are statistically significantly higher than the ones for the samples without HA (samples 6 and 9), regardless of the alginate content.

To summarize, as for the effect of the crosslinking agent on the biocompatibility of films, crosslinking by Ca^2+^ does not cause the cytotoxicity of the developed material. Previously, it was shown that the crosslinking of methylcellulose-SA films by FeCl_3_×6H_2_O, FeCl_2_×4H_2_O, FeSO_4_×7H_2_O and (NH_4_)_2_Fe(SO_4_)_2_×6H_2_O salts resulted in acute cytotoxicity of samples [74], while iron(III)-salicylic acid complex had the least cytotoxic effect. Perhaps this is due to a high concentration of crosslinking ion (Fe) (4–8 wt %) [74]. In the present study, crosslinking was made in the 5 wt % CaCl_2_ solution.

## 4. Conclusions

The PVP-HA in situ and PVP-HA ex situ composites were firstly synthesized. The structure, composition and physicochemical properties of these composite materials were investigated. NMR and FTIR spectra confirmed the presence of PVP and HA in the samples’ composition at room temperature. During thermal treatment, PVP was completely decomposed starting from 250 °C. This fact was demonstrated by TGA and X-ray analysis after temperature treatment at 400 °C and FTIR investigation of samples after 900 °C treatment.

As for the structure of bulk composites, the PVP-HA in situ sample was characterized by both spherical nanoparticles with a diameter of 5–10 nm and needle-like particles with a thickness of several nm and a length of 20 nm. The PVP-HA ex situ sample contained plate particles that were sized at 10–20 µm.

Due to the instability of PVP in aqueous medium, SA was added, and PVP-SA-HA composites were crosslinked with calcium ions. The crosslinking of the PVP-SA blend is more effective than the crosslinking of pure alginate. The interpenetrating networks of crosslinked PVP-SA permitted the material to remain stable for at least 5 days.

The obtained swelling data showed that the crosslinked composite PVP-SA-HA films exhibit hydrogel swelling characteristics. After immersion in the saline solution, a three times mass increase was detected and was maintained for 21 days of the experiment.

PVP-SA-HA in situ composite hydrogel films exhibited acute cytotoxicity for mesenchymal dental pulp stem cells, whereas PVP-SA-HA ex situ films increased the viability of DPSCs, regardless of the alginate content. The crosslinking with calcium ions did not affect the cytotoxicity of PVP-SA-HA films. Therefore, these latter developed hydrogel films are recommended for such biomedical applications, such as membranes in osteoplastic surgery and wound dressing.

## Figures and Tables

**Table 1:** Materials based on PVP with SA and HA.

Sample Number	Sample Designation	Sample Composition	Polymer Concentration in Polymer-HA * Mixture, wt %	HA/Polymer Ratio (Mass)
1	PVP *-HA	PVP, HA in situ	2.76	0.028
2	PVP-HA	PVP, HA in situ	5.52	0.014
3	PVP-HA	PVP, HA in situ	11.00	0.007
4	PVP-HA	PVP, HA ex situ	11.00	0.007
5	PVP-HA	PVP, HA ex situ	11.00	0.028
**Films for Swelling and In Vitro Investigations**				
6	PVP-SA *	PVP:SA = 1:1	5.52	-
7	PVP-SA-HA	PVP:SA = 1:1, HA in situ	5.52	0.014
8	PVP-SA-HA	PVP:SA = 1:1, HA ex situ	5.52	0.014
9	PVP-SA	PVP:SA = 1:2	5.52	-
10	PVP-SA-HA	PVP:SA = 1:2, HA in situ	5.52	0.014
11	PVP-SA-HA	PVP:SA = 1:2, HA ex situ	5.52	0.014

* HA—hydroxyapatite; PVP—polyvinylpyrrolidone; SA—sodium alginate.

**Table 2:** Dimensional characteristics of powders obtained from the PVP-HA composites (samples 1–3, Table 1) after heat treatment at T = 400 °C, determined by TEM, BET and XRD methods.

Sample	Polymer Concentration in PVP *-HA * Mixture, wt %	PVP/HA Ratio, wt %	Specific Surface Area (BET) *, m^2^/g	CSR *, nm	Particles Size from TEM Data, nm
1	2.76	0.028	16.7	17.7	30
2	5.52	0.014	19	19.6	33
3	11.00	0.07	37	17.9	45
HA	0	-	18	31.7	35

* PVP—polyvinylpyrrolidone; HA—hydroxyapatite; BET—Brunauer–Emmett–Teller specific surface area method; CSR—coherent scattering region.

## Data Availability

The experimental data on the results reported in this manuscript are available upon an official request to corresponding authors.
